# On the Existential Road From Regret to Heroism: Searching for Meaning in Life

**DOI:** 10.3389/fpsyg.2018.02375

**Published:** 2018-12-03

**Authors:** Eric R. Igou, Wijnand A. P. van Tilburg, Elaine L. Kinsella, Laura K. Buckley

**Affiliations:** ^1^Advancing Social Cognition Lab, Department of Psychology, Health Research Institute, University of Limerick, Limerick, Ireland; ^2^Psychology Department, King’s College London, London, United Kingdom; ^3^RISE Lab, Department of Psychology, Centre for Social Issues Research Institute, Health Research Institute, University of Limerick, Limerick, Ireland; ^4^Department of Psychology, University of Limerick, Limerick, Ireland

**Keywords:** regret, heroism, heroes, meaning, self-enhancement, self-regulation, existentialism

## Abstract

We investigated whether regret predicted the motivation to act heroically. In a series of studies, we examined the relationship between regret, search for meaning in life, and heroism motivation. First, Study 1 (a and b) investigated the association between regret and search for meaning in life, considering regret as a whole, action regret, and inaction regret. As expected, regret correlated positively with search for meaning in life. In two additional studies (Study 2 and 3), we examined whether regret predicted heroism motivation and whether this effect was mediated through search for meaning in life. Study 2 confirmed this hypothesis for individual differences in regret, using a correlational design. Study 3 confirmed the hypothesis for temporary experiences of regret, using an experimental design. In addition, in Study 3 we found that heroism motivation was stronger for people with high self-enhancement needs than for those with lower self-enhancement needs. We discuss the relationship between regret and heroism in light of these results and explore their implications.

## Introduction

Can regrets make us better people? Can they bring out the best in us by guiding us to heroic actions? How could that be possible given that regret is a negative experience (e.g., Gilovich and Medvec, 1995; Roese, 1997; Coricelli and Rustichini, 2010)? In the present research, inspired by those questions, we empirically examined whether behavioral intentions linked to heroism can be a function of regret. We argue that regret is associated with psychological processes that facilitate heroism motivation. Specifically, we focus on the role of search for meaning in life and consider the role of self-enhancement.

### Regret, Life, and Meaning

Humans have the need and the ability to make sense of their actions and behavior (e.g., Frankl, 1946; Postman and Weingartner, 1969; Bruner, 1990; Baumeister and Vohs, 2002; Heine et al., 2006; Van Tilburg and Igou, 2011b). Such interpretations are not always pleasant; in fact, they can be very unpleasant. People can, for example, feel embarrassed, ashamed, or guilty. Here we zero-in on one potential response: regret.

Regret is a negative experience concerning the cause and a desire to reverse the current situation (Gilovich and Medvec, 1995; Roese, 1997). It is an emotion oriented toward the past, signaling an unfavorable evaluation of a past choice (e.g., Zeelenberg et al., 1998b). Essentially, regret experiences involve thoughts about counterfactuals, that is, “what might have been” instead of “what is” (e.g., Kahneman and Miller, 1986; Connolly and Zeelenberg, 2002); these are thoughts of one’s previous action or inaction and how things would have been different, had one behaved differently (e.g., Roese, 1994, 1997; Gilovich et al., 1998; Roese and Summerville, 2005; Epstude and Roese, 2008; Epstude and Jonas, 2015; Roese and Epstude, 2017; for an overview see Mandel et al., 2007).

Regret is an experience that is crucial in the lives of humans (e.g., Stewart and Vandewater, 1999; Wrosch and Heckhausen, 2002; Timmer et al., 2005), which is reflected in the various areas where regret has been documented (for an overview see Roese and Summerville, 2005) such as health and well-being (e.g., Lecci et al., 1994; Stewart and Vandewater, 1999; Jokisaari, 2003; Connolly and Reb, 2005b; Epstude and Jonas, 2015), personality (e.g., Schwartz et al., 2002), and romance (e.g., Roese and Summerville, 2005; Timmer et al., 2005). Regrets can be very intense (e.g., Beike et al., 2009), with some people being more vulnerable to experiencing this emotion than others (e.g., Schwartz et al., 2002). Regrets can have a variety of consequences, such as self-blame (e.g., Connolly and Zeelenberg, 2002), change of expectations (e.g., Connolly and Reb, 2005a), rumination about lost opportunities (e.g., Stewart and Vandewater, 1999; Beike et al., 2009), as well as adjustments and behavior changes (e.g., Lecci et al., 1994; Roese, 1994; Roese and Summerville, 2005; Saffrey et al., 2008). When people regret something, they are likely to consider the opportunities that they did not take and the choices with better outcomes that they could have made. However, the consequences following regret need not always be negative.

Research shows there are some improvement benefits of regret that are rooted in the counterfactual thoughts associated with this experience (e.g., Markman et al., 2008; Coricelli and Rustichini, 2010), at least when people perceive some level of personal responsibility for their actions or inactions (e.g., Zeelenberg et al., 1998a). Regret involves an inconsistency between subjectively relevant goals regarding a particular situation or life in general and one’s action or inaction. Through this inconsistency, regret helps people to learn from the past. Consistent with this notion, a study by Roese and Summerville (2005) highlights that humans recognize regret as a positive influence on future behavior. Regret signals that something has gone wrong and that something needs to change. Regret can trigger a behavioral response to improve circumstances and one’s life. Indeed, regret can lead to instrumental corrective actions (e.g., Connolly and Reb, 2005a) and promote psychological adjustment (e.g., Lecci et al., 1994; Zeelenberg and Pieters, 2007; Saffrey et al., 2008), and changes in life (e.g., Lecci et al., 1994; Stewart and Vandewater, 1999; Zeelenberg, 1999; Beike et al., 2009).

The literature thus indicates that regret can lead to change. It plays a role in shaping learning processes from past experiences to the present and the future. The emotion itself is the negative sting that seems to motivate learning and change via inferences and expectations (e.g., Saffrey et al., 2008). The sting is an affective expression of the perceived inconsistency between one’s actions or inactions and one’s subjectively relevant goals, thus an inconsistency in people’s sense of meaning (e.g., Heine et al., 2006). Understanding and resolving such inconsistencies shapes these meaning frameworks and contributes to a general sense of meaning. The motivated process to learn from and resolve the inconsistencies associated with regret is essentially a search for meaning (e.g., Steger et al., 2006).

#### Search for Meaning

Recently, research on meaning making processes and their motivational components (e.g., Baumeister and Vohs, 2002; Heine et al., 2006; Van Tilburg and Igou, 2018) found that people search for meaning in the face of threats to their meaning systems (Heine et al., 2006; Steger et al., 2006). These threats can be of an affective nature, such as boredom (e.g., Van Tilburg and Igou, 2012, 2017a) or disillusionment (e.g., Maher et al., 2018). The cognitive process of meaning search follows the general need to arrive at greater insights into one’s goal and the functioning of the world (e.g., Steger et al., 2006; Van Tilburg et al., 2013). The search process is thus a motivated cognitive process directed at sources of meaning with the goal to gain more meaning. Consistently, search for meaning increases social identification with others (e.g., Van Tilburg and Igou, 2012), nostalgia (Van Tilburg et al., 2013), reliance on political ideologies (e.g., Van Tilburg and Igou, 2016; Maher et al., 2018), and inspiration by heroes (e.g., Coughlan et al., 2017); each process auguring a greater sense of meaning. In short, we pose that regret is an affective experience that is associated with particular challenges to one’s general sense of meaning, triggering a search for meaning.

### Prospects of Meaning: Heroes and Heroism

Heroes and heroism have been essential parts of human civilization as, for example, reflected in the heroic figures in ancient mythologies (for an overview see Campbell, 2004). In recent years, research has given considerable attention to the natures of heroes and heroism, highlighting the impact of heroes at the levels of groups and individuals (e.g., Sullivan and Venter, 2005, 2010; Allison and Goethals, 2011, 2013; Kinsella et al., 2015b; 2017c; Allison et al., 2017). For example, different types of heroes have been distinguished (e.g., Allison and Goethals, 2013), prototypical characteristics of heroes have been identified (e.g., Kinsella et al., 2015b, 2017a), and important social and psychological functions served by heroes – such as enhancement, protection, and moral guidance (e.g., Kinsella et al., 2015a) – have been reported.

Heroes and heroism are positively laden concepts infused with superordinate values and admirable behaviors. Representations of heroes differ from those of role models and leaders (Kinsella et al., 2015b, 2017b). Heroes and heroism stand out with regard to the moral goals that are pursued and how they are pursued, namely with personal sacrifices and risks. Heroes and heroic acts are generally appreciated, inspiring, and comforting for groups and individuals, especially in unsatisfactory and threatening times (e.g., Allison and Goethals, 2011). Beliefs about heroes and heroism are widely shared and central in cultures and individuals’ lives (e.g., Kinsella et al., 2015b; Bronk and Brian, 2016).

In line with this notion, heroes and heroism are sources of meaning and inspiration (e.g., Früchtl, 2009; Bronk and Brian, 2016; Green et al., 2017; Kinsella et al., 2017a), and search for meaning can thus be linked to perceptions of heroes and heroism and associated inspiration. We pose that this process is especially relevant when the “need” for heroes or heroism is relatively pronounced. For example, Coughlan et al. (2017) illustrate this for boredom. Specifically, boredom reflects meaninglessness of one’s activities or even life in general (e.g., Van Tilburg and Igou, 2011a, 2012, 2016, 2017a,b). Coughlan et al. argued and found that people who are prone to boredom hold more positive perceptions of cultural heroes (e.g., Dr. Marin Luther King Jr.), namely how special and inspiring the person was, how much they admired the person, and much the person was of personal significance and purpose. Importantly, the association between boredom proneness and these hero perceptions was mediated by search for meaning in life. That is, boredom – an experience that motivates a search for meaning (Van Tilburg and Igou, 2011a, 2012) – predicted greater appreciation of heroes to the extent that boredom involved a meaning search. The proposition that the perception of heroes and heroism can serve as a response to threatening experiences is not limited to boredom; we propose that regret is associated with a challenge to people’s meaning system: people feel regret because their past actions or inactions are inconsistent with their goals. Regret motivates a cognitive process that helps making sense of the situation and oneself. Given that heroes and heroism are sources of meaning, we thus reasoned that experiences of regret would transfer into inspiration and show the readiness to act heroically via a search for meaning in life.

### Study Overview

Study 1 examined the proposed link between regret and search for meaning. Specifically, Study 1a examined the association between individual differences in regret and search for meaning in life; Study 1b examined individual differences in the general form of regret, action regret, and inaction regret, and search for meaning in life. Study 2 adopted a correlational design to test at an individual difference level the effects of regret on the motivation to be heroic via search for meaning in life. Study 3 adopted an experimental design to test the effects of regret on heroism motivation via meaning search. In addition, we examined the effects of individual differences in self-enhancement needs on the relationship between regret and heroism motivation.

## Study 1 (A-B): Regret and Meaning Search

We pose that regret is at least partly associated with existential processes, in that it leads people to search for meaning in life. Study 1 was designed to test if regret predicted search for meaning in life. We examined whether this process occurred for two forms of regret often discussed in the literature, namely action and inaction regret (e.g., Gilovich et al., 1998). We predicted that regret would be associated with an increase in search for meaning in life. Given that our approach makes no assumptions about the differences between actions and inactions, we expected the proposed effect of regret on meaning search for both forms of regret.

### Materials and Methods

#### Participants and Design

Study 1a investigated the association between individual differences in regret and search for meaning using a correlational design. We recruited 53 participants via the online portal Mechanical Turk (MTurk ^1^; in the United States and in India). One participant was excluded because of missing data, resulting in a total of 52 participants (32 male, 20 female; *M_age_* = 35.9 years; 43 US American, 9 Indian). Study 1b had a correlational design measuring general regret, action regret, inaction regret and meaning search. We recruited 156 participants residing in the United States on MTurk. Two participants were excluded because they were extreme outliers in time spent on the questionnaire, leaving a total of 154 participants (90 female, 64 male; *M_age_* = 34.9).

#### Materials and Procedure

We programmed the studies using the online computer survey program Questback; data were collected using the MTurk recruitment platform. After signing consent forms, participants reported demographic information.

In Study 1a, we next administered the five-item regret scale (e.g., *When I think about how I’m doing in life, I often assess opportunities I have passed up*; α = 0.77; Schwartz et al., 2002), ranging from 1 (*completely disagree*) to 7 (*completely agree*), and the meaning in life questionnaire (MLQ; Steger et al., 2006), with the two five-item subscales measuring search for meaning in life (e.g., *I am seeking a purpose or mission in my life;* α = 0.96) and presence of meaning in life (α = 0.96), using scales from 1 (*absolutely untrue*) to 7 (*absolutely true*). To control for a general form of affect, we included the four-item subjective happiness scale (Lyubomirsky and Lepper, 1999; α = 0.75; using seven-point scales, see Supplementary Materials details), and for exploratory reasons we included the 20-item desirability of control scale (Burger and Cooper, 1979; α = 0.75) ranging from 1 (*statement does not apply to me*) to 7 (*statement always applied to me*)^2^. The scales were presented in this order: desirability of control scale, regret scale, MLQ, global subjective happiness scale.

In Study 1b, we administered four-item measures of action and inaction regret (α = 0.93 for each scale): *How prone are you to feeling regret about an action (inaction)?; How often do you experience regret about an action (inaction)?; Generally speaking, how often do you feel regret about an action (inaction)?* (1 = *not at all*/*never*, 7 = *very much*/*all the time*); *Specifically, how often do you feel regret?* (1 = *once or twice a year*, 7 = *at least once a day*). We then administered the regret scale (α = 0.82; Schwartz et al., 2002) and subsequently the search for and presence of meaning in life scales (both α = 0.94) of the MLQ (Steger et al., 2006)^3^.

Afterward, participants in both studies were debriefed and rewarded with €0.50 for their participation.

### Results and Discussion

In Study 1a, regret correlated with search for meaning in life (*r* = 0.50, *p* < 0.001). Regret did not correlate significantly with presence of meaning in life (*r* = -0.14, *p* = 0.31). Search for and presence of meaning in life did not correlate significantly with each other (*r* = -0.20, *p* = 0.15). Happiness correlated with regret negatively (*r* = -0.46, *p* < 0.001) and with presence of meaning in life positively (*r* = 0.67, *p* < 0.001). When we conducted a partial correlation with regret and search for meaning in life while controlling for happiness, we still observed the predicted correlation (*r* = 0.48, *p* < 0.001). This result confirmed our prediction that higher (vs. lower) levels of regret were associated with higher (vs. lower) levels of search for meaning in life. No other correlations were significant^4^^,^^5^ .

Replicating results of Study 1a, in Study 1b we found that regret scale scores correlated with search for meaning in life scores (*r* = 0.40, *p* < 0.001). We also found positive associations between search for meaning in life and action regret (*r* = 0.23, *p* = 0.004) and inaction regret (*r* = 0.20, *p* = 0.014), in particular. In addition, presence of meaning in life correlated negatively with search for meaning in life (*r* = -0.26, *p* = 0.001). Meaning presence also correlated negatively with all regret measures, the regret scale (*r* = -0.31, *p* < 0.001), action regret (*r* = -0.23, *p* = 0.005), and inaction regret (*r* = -0.17, *p* = 0.03), indicating a weak negative association between meaning presence and different forms of regret^6^.

In sum, the results of Study 1 (a and b) demonstrate that regret is associated with search for meaning in life. Specifically, higher levels of regret were associated with higher levels of meaning search. Addressing the distinction in the literature between action and inaction regret, our results show that both of these forms of regret are associated with search for meaning in life. These results support the hypothesis that regret has existential qualities by being associated with search for meaning in life. Although not central to our examination, we note rather inconsistent effects across Studies 1a and 1b for the association between regret and presence of meaning in life. Presumably, this speaks to the nature of regret as a complex human experience that indicates reduced purpose in one’s behavior but at the same time communicates causalities and responsibilities for actions or inaction thus providing some level of epistemic meaning.

The following studies examined the relationship between regret and heroism motivation, and the predicted mediating role of search for meaning in life.

## Study 2: Individual Differences in Regret and Heroism Motivation

Study 2 tested whether individual differences in regret are associated with the motivation to act heroically, and whether this association would be mediated by search for meaning in life. Essentially, we propose that people prone to regret experiences search for meaning in life, and that this search in turn predicts the motivation to engage in heroic activities, a source of meaning (e.g., Kinsella et al., 2017b; Coughlan et al., 2017). We included a measurement for people’s mood to control for people’s affective state as a variable that could theoretically account for the proposed regret effects on meaning search and heroism motivation.

### Materials and Methods

#### Participants and Design

We recruited 122 participants residing in the United States on MTurk. Due to non-completion and missing data of some participants, 11 participants were excluded from the data set, resulting in a total of 111 participants (62 female, 49 male, 3 unspecified; *M_age_* = 38.14; 106 US Americans, 1 Canadian, 1 British, 1 Irish, 1 Montenegrin, 1 unspecified). For this correlational study, we rewarded participants with $0.40.

#### Procedure and Materials

After providing informed consent, participants reported demographic information (ethnicity, gender, age). Next, they worked on two items measuring participants’ mood (*r* = 0.94, *p* < 0.001), *How is your mood?* measured on a scale from 1 (*very bad*) to 7 (*very good*), and *How do you feel?* measured on a scale from 1 (*very sad*) to 7 (*very happy*). Participants then filled out the regret scale (α = 0.82; Schwartz et al., 2002; Study 1) and then the search for meaning in life scale (Steger et al., 2006; Study 1) as well as two items with using the identical scale that relate to search for meaningful activities, *I am always looking to do things that are meaningful* and *I am seeking to do things that have meaning for me and others*. We reasoned that adding items on activities would be adequate in the context of heroic activities. The resultant seven-item scale was highly reliable (α = 0.95).

Next, participants completed a four-item measure of heroism motivation (α = 0.90). Specifically, they were instructed to think about their life, who they want to be, what they want to be known for. They then indicated their agreement to the statements, *I want to behave heroically towards others if it is necessary and I have the opportunity to do so; It is important to me to be seen as someone who can act heroically; I strive to be a hero for other people, if the situation requires someone to step up; It is significant to my life to be seen as someone who has the qualities of a hero*, on scales from 1 (*not at all*) to 5 (*very much*). Afterward, participants were thanked, debriefed, and rewarded.

### Results and Discussion

Regret correlated with search for meaning in life (*r* = 0.35, *p* < 0.001) and with heroism motivation (*r* = 0.21, *p* = 0.025). Meaning search correlated with heroism motivation (*r* = 0.31, *p* = 0.001). Mood correlated negatively with regret (*r* = -0.28, *p* = 0.003) and was not significantly correlated with search for meaning (*r* = -0.06, *p* = 0.520).

**FIGURE 1:**
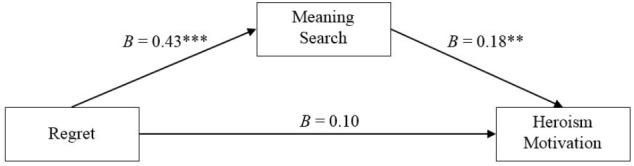
Mediation model of regret, meaning search, and heroism motivation (Study 2). ^∗^*p* ≤ 0.05, ^∗∗^*p* ≤ 0.01, ^∗∗∗^*p* ≤ 0.001; indirect effect of regret on heroism motivation through meaning search: *B* = 0.08, *SE* = 0.04, 95% CI [0.007, 0.174].

We proceeded to examine the indirect association between regret and heroism motivation via search for meaning. To estimate this, we used PROCESS (Version 3; Hayes, 2018), Model 4 (10,000 bootstraps), where regret was entered as the predictor, meaning search as the mediator, and heroism motivation as the criterion. Regret had a total effect on heroism motivation, *B* = 0.18, *SE* = 0.08, *t*(109) = 2.27, *p* = 0.025, 95% Cl [0.022, 0.328], but there was no significant direct effect, *B* = 0.10, *SE* = 0.08, *t*(109) = 1.20, *p* = 0.23, 95% Cl [-0.063, 0.254]. Most importantly, we observed the predicted indirect effect of regret on heroism motivation though meaning search even when controlling for mood by adding the measure as a covariate, *B* = 0.08, *SE* = 0.04, 95% Cl [0.009, 0.163] (Figure 1). When we added mood as a covariate to the analysis, regret had a total effect on heroism motivation of *B* = 0.27, *SE* = 0.07, *t*(108) = 3.59, *p* < 0.001, 95% Cl [0.119, 0.413] and a direct effect on heroism motivation of *B* = 0.19, *SE* = 0.08, *t*(108) = 2.47, *p* = 0.01, 95% Cl [0.038, 0.342]. The covariate, mood, had a direct effect on heroism motivation, *B* = 0.33, *SE* = 0.07, *t*(107) = 4.46, *p* < 0.001, 95% Cl [0.182, 0.472]. Most importantly, we observed the predicted indirect effect of regret on heroism motivation though meaning search even when controlling for mood by adding the measure as a covariate, *B* = 0.8, *SE* = 0.04, 95% Cl [0.009, 0.164].

Individual differences in the motivation to act heroically were associated with individual differences in regret. In part, this relationship was plausibly due to people’s search for meaning in life, as predicted. People’s mood was related to regret and to heroism motivation, however, these associations were unrelated to the focal test in question. In the following study, we examined the relationship between the temporary experience of regret on heroism motivation and the predicted mediating role of search for meaning in life.

## Study 3: Temporary Regret Experiences and Heroism Motivation

The goal of Study 3 was to examine the causal relationship between regret and people’s motivation to act heroically. Given that regret promotes a search for meaning in life and that heroism is a source of meaning, we argue that regret increases the motivation to engage in heroic acts. We examined this hypothesis experimentally by manipulating regret and measuring heroism motivation.

In addition, we examined whether the effect could be explained by self-enhancement needs. Needs for self-enhancement and motivated actions serving them are central in humans (for an overview see Alicke and Sedikides, 2010). More specifically, our hypothesis rests on the finding that heroes serve particular social and psychological functions listed by Kinsella et al. (2015a), who argue that heroes have a protective function, give moral guidance, and serve an enhancement function. We interpret enhancement in the context of heroism motivation as the strategy to enhance the self via heroic actions. This argument is consistent with the literature on pro-social behavior, which reports that at least some pro-social acts serve self-enhancement needs (e.g., Batson, 1987). Self-enhancement has several components (e.g., Hepper et al., 2010), and we focused in particular on people’s strategies to construe situations favorably and to affirm the self, especially when encountering challenges to the self. These strategies match the challenge of regret experiences and are functional for the engagement with the social environment via rather extreme pro-social activities such as heroism (e.g., Franco et al., 2011). We thus added an individual difference measure of self-enhancement to examine whether heroism motivation increased when experiencing regret in particular for participants with a high need for self-enhancement compared to those with a low need for self-enhancement.

### Materials and Methods

#### Participants and Design

We recruited 255 participants through MTurk. Due to non-completion and missing data, 25 participants were excluded from the data set, resulting in a total of 230 (*female* = 127, *male =* 100, *other =* 3; *M_age_* = 39.7). We experimentally manipulated regret and then measured heroism motivation, meaning search, and self-enhancement; accordingly, participants were randomly assigned to either the regret condition or the control condition. They were rewarded $0.41 for their participation.

#### Procedure and Materials

After providing informed consent, participants reported their demographics. Then they worked on the regret induction task and the manipulation check items. We induced regret using an autobiographical recall procedure (e.g., Lerner and Keltner, 2001; Martinez and Zeelenberg, 2015). This procedure is based on the notion that remembering particular episodes activates the affective experiences that are associated with these memories. Specifically, in the experimental condition, in a box appearing on the screen, participants described a situation when they experienced ‘the biggest regret in their life’ in at least three sentences so that others would be able to understand the experience. We then asked them to report the physical reaction to the situation and how it felt in a second box that appeared on the screen. In the control condition, participants described an “everyday life experience when nothing special happened, a day with mundane activities and events” in the first box and the physical reactions and how they felt in the second box. All participants then completed manipulation check items (*r* = 0.93, *p* < 0.001), *By thinking of the situation that I just described, feelings of regret arise in me* and *How much regret do you feel right now?* Responding on scales from 1 *(not at all)* to 7 *(very much)*. Then, we presented two filler items unrelated to the research questions in order to reduce the likelihood of demand characteristics in response to the focal questions. We asked for the liking of the color and shape of a car (Citroën C4 Cactus) using scales from 1 (*not at all*) to 7 (*very much*).

Afterward, participants responded to the seven items measuring acute search for meaning in life based on the measure used in Study 2 (e.g., *Right now, I feel like looking for something that would make my life meaningful*; *At this moment, I feel like seeking a purpose or mission in my life*). The resultant scale was highly reliable (α = 0.94). Following these items, participants worked on a self-enhancement measure suitable for this study. We used the brief self-enhancement and self-protection scale (Hepper et al., 2010), adapting items from the two subscales that measure *favorable construal* and *self-affirming reflections* (α = 0.93). Participants indicated their agreement on a scale from 1 (*low*) to 6 (*high*) to items such as, “*Looking back, I believe that I have been changing, growing, and improving as a person*,” “*I am aware of my values and what matters to me*.” We did not include items of the other two subscales, positivity embracement and defensiveness, as we deemed them rather secondary in the context of heroism (see Supplementary Materials for the measure).

Next, participants completed the heroism motivation measure (α = 0.93; see Study 2). Afterward, they were thanked, debriefed, and rewarded.

### Results and Discussion

Participants in the regret condition felt more regret than participants in the control condition (*M* = 5.67, *SD* = 1.37 vs. *M* = 1.89, *SD* = 1.44), *t*(228) = 20.40, *p* < 0.001, *d* = 1.80, indicating that the manipulation was successful. Participants with higher levels of regret indicated a stronger motivation to search for meaning in life than participants in the control condition (*M* = 4.61, *SD* = 1.65 vs. *M* = 4.13, *SD* = 1.69), *t*(228) = 2.16, *p* = 0.03, *d* = 0.29. Participants in the regret condition indicated a higher level of heroism motivation than participants in the control condition, however, this difference was only marginally significant (*M* = 3.50, *SD* = 1.07 vs. *M* = 3.23, *SD* = 1.09), *t*(228) = 1.88, *p* = 0.06, *d* = 0.25. Search for meaning in life was positively correlated with heroism motivation (*r* = 0.35, *p* < 0.001). Individual difference in self-enhancement were unaffected by regret (*t* > 1), uncorrelated with search for meaning (*r* = -0.03, *p* = 0.69) but correlated positively with heroism motivation (*r* = 0.25, *p* < 0.001).

**FIGURE 2:**
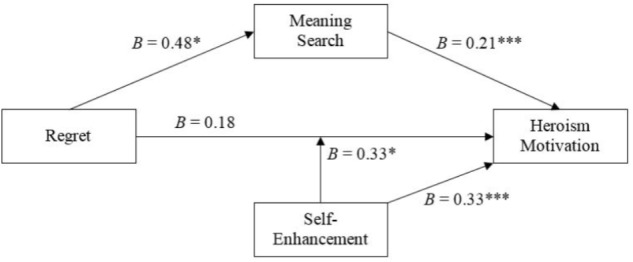
Integrated mediation and moderation model of regret, meaning search, self-enhancement, and heroism motivation (Study 3). ^∗^*p* ≤ 0.05, ^∗∗^*p* ≤ 0.01, ^∗∗∗^*p* ≤ 0.001; indirect effect of regret on heroism motivation through meaning search: *B* = 0.10, *SE* = 0.05, 95% CI [0.008, 0.212]; condition effect of regret and self-enhancement on heroism motivation: *B* = 0.33, *SE* = 0.16, 95% CI [0.011, 0.652].

In the next step, we examined the indirect effect of regret on heroism via search for meaning and the moderating role of self-enhancement. For this, we used PROCESS (Version 3; Hayes, 2018), Model 5 (10,000 bootstraps), which tested the mediational effect of regret on heroism motivation via search for meaning in life and the moderating effect of self-enhancement on heroism motivation in conjunction with regret (Figure 2). We found that regret affected heroism motivation via search for meaning in life, *B* = 0.10, *SE* = 0.05, 95% Cl [0.008, 0.212]. In addition, independently of the mediation effect by meaning search, regret affected heroism motivation more strongly for participants with high self-enhancement needs, *B* = 0.49, *SE* = 0.20, *t*(225) = 2.44, *p* = 0.02, 95% Cl [0.095, 0.885], than for participants with medium and low self-enhancement needs, *B* = 0.22, *SE* = 0.13, *t*(225) = 1.70, *p* = 0.09, 95% Cl [-0.036, 0.485] and *B* = -0.11, *SE* = 0.19, *t* = -0.56, *p* = 0.58, 95% [-0.484, 0.270], indicated by a significant interaction of regret and self-enhancement, *B* = 0.33, *SE* = 0.16, *t*(225) = 2.04, *p* = 0.043, 95% CL [0.011, 0.652].

In sum, these results demonstrate that regret affects heroism motivation, but that this effect has two important characteristics. Crucial for the overarching hypothesis of the current work, these heroism effects are partly explained by people’s search for meaning in life. The second characteristic is that regret can also affect heroism motivation directly, for people who have high needs for self-enhancement, specifically favorable construals of situations and self-affirming reflections.

## General Discussion

We tested if regret increases the motivation to engage in heroic activities. We argue that, in part, regret is an existential experience in that it motivates people to search for meaning in life. We pose that it is, in part, this existential process that is responsible for people’s heroism motivation in response to regret.

In Study 1a and 1b, stronger regret was associated with elevated search for meaning in life. This association held for action regret as well as for inaction regret after controlling for general happiness. Study 2 went beyond Study 1 by examining the association between regret, search for meaning in life, and heroism motivation. As predicted, individual differences in regret predicted individual differences in heroism motivation. Importantly, this effect was mediated by search for meaning in life. This mediational effect remained reliable after controlling for participants’ mood state. Study 3 went beyond Study 2 by examining temporary experiences of regret on search for meaning in life and heroism motivation, and by also considering self-enhancement needs and their effect on heroism motivation. As predicted, temporary regret affected heroism motivation through search for meaning in life. We also found that participants with high self-enhancement needs were more motivated to act heroically than those with low self-enhancement needs and that this was particularly the case when participants experienced regret. Importantly, the existential road from regret to heroism motivation was independent of participants self-enhancement needs.

### Heroism

In recent years, researchers from different academic areas have examined the characteristic of heroes, the functions of heroes, and conditions of heroism (for an overview see Allison et al., 2017). Our research examined the motivation to act heroically as a function of an important experience in life: regret. The results demonstrate that the negative experience of regret can foster heroism motivation, thus increasing the likelihood for people to display heroic actions. In that sense, a negative experience increases the chances of pro-social outcomes. Our research supports the hypothesis that regret experiences leave people with a need to find meaning in their lives and that behaving heroically provides an opportunity to re-establish meaning. This is consistent with the notion that heroism is a source of meaning, potentially playing a role when people regulate their sense of meaning (e.g., Coughlan et al., 2017; Green et al., 2017; Kinsella et al., 2017b). Our research also shows that people who have high self-enhancement needs, in our case those with a strong inclination to construe situations favorably and to affirm the self, are relatively motivated to act heroically when experiencing regret (Study 3). We also found that individual differences in mood predicted heroism motivation, such that people with higher positive mood were more motivated in this regard (Study 2). Taken together, our research makes an important contribution to the literature on transformations of life in form of hero journeys that people may undertake (e.g., Allison and Goethals, 2011) and the ongoing pursuit of understanding how the person and the situation contribute to heroism (e.g., Bronk and Brian, 2016).

### Regret and Existentialism

Regret experiences are important and functional for actions (e.g., Roese and Epstude, 2017; Zeelenberg and Pieters, 2007) and life in general (Stewart and Vandewater, 1999; Timmer et al., 2005). Importantly, regret facilitates learning processes where perceived mistakes or wrongdoings of the past can be avoided and behavior can be improved (e.g., Zeelenberg, 1999; Zeelenberg and Pieters, 2007; Coricelli and Rustichini, 2010). Our studies add to this notion the existential component of searching for meaning in life, a motivation that is directed to establish a higher sense of meaning. We believe that is it crucial to consider this psychological variable, as it has been shown to be central in regulating meaning (e.g., Bruner, 1990; Steger et al., 2006). The robust relationship we found for individual differences in regret and meaning search as well as for temporary experiences of regret and meaning search enables a more precise process-oriented perspective on the potential consequences of regret, when search for meaning in life is likely to be involved.

### Limitations and Future Research

We acknowledge that our studies have a number of limitations. The link to heroism that we examined is constrained by an explicit, self-reported motivation to engage in heroic activities and to be seen as heroic by others. Although it would have been ideal to test the effects with a range of heroism measures, we found the effects of regret on heroism motivation via search for meaning in life seem to be robust across two studies (Study 2 and 3). Future research needs to include other measures of motivations to act heroically and heroism, such as the inspiration by heroes (e.g., Coughlan et al., 2017) and actual heroic behavior. The heroism motivation that we measured could serve as a crucial process variable when examining heroic action or the effects of inspiration by heroes.

With regard to actual behavior, we are cautious as to whether search for meaning in life would have a strong effect in a complex social environment. It might be that other variables such as self-enhancement may need to be present in order to evoke the effect of regret on actual heroism. Study 3 might give an idea on how multiple variables could be involved and related. We note that the experimental induction in Study 3 is limited by potentially raising particular issues of past experiences in the experimental and/or the control conditions. Relatedly, in this study, we did not control for additional affective experiences that might have been activated by the induction procedure.

Our studies focused on *search for* *meanin*g in life as the crucial mediating variable that explains at least in part the relationship between regret and heroism motivation. In this respect, regret qualifies as an affective experience that initiates an existential process. The regret experience itself is based on perceived inconsistencies within one’s meaning frameworks, second-guessing one’s action or inaction by considering alternative courses of action that may have been more adequate. It would thus be conceivable that dwelling on regrets is associated with a reduced sense of meaning in life. Although not central to our examination, our results do not suggest that regret experiences profoundly reduce a sense of meaning in life. It might be more complicated. Possibly, lack of meaning is more strongly associated with the cognitive foundation of regret than with the experience itself. Or, two different qualities of meaning may need to be considered. Teleological meaning refers to the purpose of meaning in life, while epistemic meaning refers to the general understanding of situations, the self, others, and the world in general (e.g., Heine et al., 2006; Van Tilburg and Igou, 2011b, 2013). It is possible that regret experiences materialize because of an increased understanding that one’s actions or inactions diverted from one’s goals and objectives and that alternative actions or inactions were available. In that sense, epistemic meaning might be provided, at least to some degree. However, regret experiences raise the issue of falling short compared to one’s important goals in life. This discrepancy might well reduce a perceived *purpose* in life. Future research should examine how regret affects epistemic vs. teleological meaning and whether the meaning search processes that we highlight are responses to threats of one or both of these meaning frameworks.

Although our research focuses on search for meaning in life as a mediating variable between regret experiences and heroism motivation, we do not rule out consequences of regret experiences that might be largely unrelated to meaning search (e.g., feeling depressed or anxious). Our research is embedded in the literature and builds in part on our previous research on existential experiences and heroes (e.g., Coughlan et al., 2017; Kinsella et al., 2017a). Additionally, accompanying psychological processes are quite possible and worthy of investigation. We propose that future research examines the causes of regret, the temporal distance of regret to heroism motivation and action, a range of mediators aside from meaning search (e.g., self-presentation, social desirability), various indicators of heroism (motivation and action), and consequences of regret-based heroism (e.g., self-esteem, perceptions of personal growth, well-being). Individual differences in a range of qualities (e.g., morality, self-enhancement, self-improvement, social desirability) seem also important, potentially functioning as moderators between regret and heroism, as the results of Study 3 suggest. In this regard, it should be noted that regret intensity seems to be associated with self-esteem contingency (i.e., instability, fragility), especially if the events involved seem controllable (Wilkinson et al., 2015). It might thus be worth examining whether, and under which conditions, self-esteem contingency can motivate people to engage in heroic acts when they experience regrets. Finally, contexts may or may not give rise to heroism. More importantly, some contexts may give rise to heroism based on particular psychological processes (e.g., search for meaning, self-enhancement, social desirability) while other contexts give rise to heroism based on different psychological processes. Future research needs to address these links in general and also with regard to the specific link between regret and heroism.

All of our studies were conducted on MTurk. Certainly, the validity of online studies is often limited due to the lack of experimental control and other technical constraints (e.g., Zhou and Fishbach, 2016). Despite these limitations, the results were consistent across a series of studies using different designs, thus supporting the notion that our studies were adequate for testing our hypotheses. However, future research should examine the link between regret and heroism using a range of procedures, such as different investigative methods (e.g., computer lab study, paper and pencil questionnaire), different recruitment procedures of participants (e.g., participant pool, volunteers), accompanied by samples from different populations of participants (cultures, socioeconomic status).

## Conclusions and Implications

This research provides important insights into both the existential nature of regret and its impact on heroism. To our knowledge, these studies provide first evidence that regret predicts search for meaning in life and that it encourages heroic behavior. Importantly, this research shows how the negative experience of regret increases motivations to engage in extreme forms of pro-social behavior, namely heroism. These results further support the notion that human beings are driven by existential motives and that heroism – a source of meaning – can result from existential motivations. This research provides the basis for further examination of different forms of heroism and heroic behavior as a function of regret experiences.

## Data Availability

The datasets collected and analyzed for this study can be found in the Open Science Framework repository. https://osf.io/c8zar.

## Ethics Statement

This study was carried out in accordance with the recommendations of “EHS Guidelines for ethics research by Education and Health Science Research Ethics Committee (EHSREC)” and the Psychiatry, Nursing, and Midwifery Research Ethics Committee Minimal Risk Route (PNM-REC Minimal Risk Route) with written informed consent from all subjects. All subjects gave written informed consent in accordance with the Declaration of Helsinki. The protocol was approved by the “Education and Health Science Research Ethics Committee” and the “Psychiatry, Nursing, and Midwifery Research Ethics Committee Minimal Risk Route.”

## Author Contributions

EI developed the rationale for this research, was involved in the design of all studies and the analyses of their results, conducted Studies 2 and 3, and wrote the manuscript. WvT contributed to the rationale of the research, conducted Study 1b, and revised the manuscript. EK contributed to the rationale of the research and revised the manuscript. LB conducted Study 1a as part of her final year project (bachelor thesis) and revised the manuscript.

## Conflict of Interest Statement

The authors declare that the research was conducted in the absence of any commercial or financial relationships that could be construed as a potential conflict of interest.

## Footnotes

1 www.mturk.com

2 Search for and presence of presence of meaning in life usually have a small negative correlations, reflecting that these variables represent largely different psychological processes of people’s meaning system (e.g., Steger et al., 2006). Given that it was conceptually unclear whether presence of meaning impacts on heroism motivation and how it related to regret (see section Limitations and Future Research), we had no clear predictions for meaning presence and heroism motivation, and thus treated meaning presence as an exploratory variable. Two of the authors discussed the potential role of the desirability of control and decided to include the measure for exploratory reasons.

3 For exploratory reasons, we added a three-item measure of nostalgia (e.g., *I am feeling quite nostalgic*; scale from 1 [*not at all*] to 6 [*very much*]; α = 0.98) at the end of the questionnaire.

4 To address potential concerns about the relatively low participant number in Study 1a, we conducted another study on MTurk with 100 participants (living in the United States; 55 female, 24 male; *M_age_* = 38.3). It consisted of a battery of scales, including the regret scale (Schwartz et al., 2002) and search for meaning in life scale (Steger et al., 2006). The positive association of regret and meaning search was replicated (*r* = 0.40, *p* < 0.001).

5 Desirability of control had no significant association with any variable (all *p*’s > 0.23).

6 Nostalgia correlated with presence of meaning in life (*r* = 0.18, *p* < 0.05), consistent with the literature (e.g., Van Tilburg et al., 2013).

## Supplementary Material

Subjective Happiness Scale (Lyubomirsky and Lepper, 1999); Self-Enhancement Needs Scale adapted from Hepper et al. (2010). https://osf.io/c8zar
